# Potential drug targets for Neuromyelitis optica spectrum disorders (NMOSD): A Mendelian randomization analysis

**DOI:** 10.1371/journal.pone.0322098

**Published:** 2025-04-28

**Authors:** Hongqi Meng, Shengnan Wang, Lulu Gu, Yuhao Wang, Beibei Li, Ruyue Lv, Letian Xue, Yanming Ren, Li Xu, Ling Mao, Peng Sun

**Affiliations:** 1 Department of Emergency Medicine, Union Hospital, Tongji Medical College, Huazhong University of Science and Technology, Wuhan, Hubei, China; 2 Key Laboratory of Anesthesiology and Resuscitation (Huazhong University of Science and Technology), Ministry of Education, Wuhan, Hubei Province, China; 3 Department of Neurology, Union Hospital, Tongji Medical College, Huazhong University of Science and Technology, Wuhan, Hubei, China; King's College Hospital NHS Trust: King's College Hospital NHS Foundation Trust, UNITED KINGDOM OF GREAT BRITAIN AND NORTHERN IRELAND

## Abstract

**Background:**

Certain peripheral proteins are involved in the development of Neuromyelitis optica spectrum disorders (NMOSD), such as IL-6, complement proteins, and MHC class II molecules. However, the roles of other new protein biomarkers are unclear. Current NMOSD treatments (e.g., intravenous pulse methylprednisolone, or satralizumab for IL-6 receptor inhibition) can only manage symptoms, necessitating the identification of new drug targets to treat NMOSD. The objective of this study is to identify potential drug targets for NMOSD through Mendelian randomization (MR) analysis, thereby addressing the limitations of current treatments and providing better clinical options for patients.

**Methods:**

NMOSD potential drug targets were evaluated via MR. Data was obtained from a genome-wide association study (GWAS) with 132 individuals with AQP4-IgG-positive NMOSD and 1244 controls. Genetic instruments for plasma and cerebrospinal fluid (CSF) proteins were identified. Sensitivity analyses were conducted using Bayesian co-localization, reverse causality testing and phenotype scanning. Additionally, a comparison and analysis of protein-protein interactions (PPI) were conducted to identify potential causal proteins. The implications of these findings were further explored by evaluating existing NMOSD drugs and their respective targets.

**Results:**

Four proteins were identified at the FDR correction via MR analysis (*p* < 0.05). Higher levels of PF4V1 (OR = 0.47; 95% CI, 0.29–0.78; *p* = 3.39 × 10^−3^) and FAM3B (OR = 0.12; 95% CI, 0.03–0.45; *p* = 1.65 × 10^−3^) were associated with a reduced risk of NMOSD, whereas elevated SERPINA1 (OR = 2.28; 95% CI, 1.29–4.04; *p*= 4.71 × 10^−3^) and CLEC11A (OR = 13.45; 95% CI, 1.29–4.04; *p* = 4.71 × 10^−3^) were related to an increased risk of NMOSD. Bayesian co-localization showed that the protein-related genes shared the same mutation as NMOSD (all PPH_4_>0.80). Reverse causality testing showed no evidence of NMOSD-driven protein changes (all *p* > 0.05). PPI analysis revealed SERPINA1 interacts with PF4V1 (combined score = 0.72). Drug evaluation identified Mercaptoethanol and Ferrous gluconate as repurposing candidates.

**Conclusion:**

Increased levels of plasma CLEC11A and SERPINA1 are correlated with an elevated risk of NMOSD, whereas elevated levels of plasma PF4V1 and CSF FAM3B are associated with a decreased risk of NMOSD. The opposing effects of risk or protective proteins suggest synergistic targeting could improve efficacy beyond current immunosuppressive regimens. Nonetheless, clinical trials are required to confirm the findings.

## Introduction

Neuromyelitis optica spectrum disorders (NMOSD) are humoral autoimmune inflammatory demyelinating diseases mostly affecting the central nervous system (CNS), including the spinal cord and optic nerves [1, 2]. NMOSD is more common in women and is related to aquaporin-4 (AQP4) antibodies. The incidence and prevalence of NMOSD peak during the middle age [3]. Epidemiological studies have shown that NMOSD is a rare disease with global prevalence rates of 0.3–4.4/100,000 individuals. Additionally, the incidence of NMOSD is significantly different across various ethnicities. Specifically, NMOSD incidence is highest among individuals of African descent, followed by those of Asian heritage, and least in White populations [4].

NMOSD is treated with various pharmacological treatments targeting different mechanisms. For instance, immune response can be attenuated by depleting B lymphocytes through antigen-antibody binding, or by inhibiting the proliferation of both T and B lymphocytes via interference with RNA or DNA synthesis, thereby modulating the immune system [5,6]. First-line NMOSD therapies include glucocorticoids, rituximab (RTX), and azathioprine (AZA), while second-line agents include methotrexate, mitoxantrone, and mycophenolate mofetil (MMF) [7,8]. Nonetheless, innovative treatment options, such as complement inhibitors (Eculizumab), IL-6 receptor antagonists (satralizumab and tocilizumab), and B-cell-targeting biologics (inebilizumab and ocrelizumab) have emerged [9,10]. These novel therapies have demonstrated promising results in clinical trials, offering alternatives for NMOSD patients.

However, the underlying pathogenesis of NMOSD is partially understood. The most common hypotheses for the pathogenesis of NMOSD include AQP4-IgG-mediated astrocyte damage, dysregulation of T and B cell functions, activation of the complement system, and blood-brain barrier disruption [6,11–14]. However, these hypotheses do not fully explain the pathogenesis of AQP4-IgG-negative cases or atypical NMOSD cases, nor do they clarify the mechanisms by which astrocyte damage leads to demyelination, the specific roles of T and B cells, or how complement activation interacts with other immune mechanisms. As a result, many existing therapies exhibit limited efficacy, often achieving only partial disease control. Over 50% of NMOSD patients struggle to attain significant systemic control. This can lead to severe complications, including chronic neuropathic pain, permanent vision impairment, paralysis, greatly reducing the quality of life or even death [15,16]. Therefore, further research should identify effective treatments and therapeutic targets for NMOSD. Besides, understanding the immunopathological mechanisms underlying NMOSD is crucial for developing new therapeutic strategies [17]. More innovative treatment options may significantly enhance patient outcomes and improve their quality of life [18].

Human proteins are vital in numerous biological processes, including growth, repair, signaling, transport, storage, and immune defense, making them essential targets for drug development. Genetic correlations connecting protein therapeutic targets to diseases can significantly improve the efficiency and success rates of clinical drug development while lowering research expenses. Genome-wide association studies (GWAS) provide new perspectives on complicated diseases and thus are promising for the identification of new drug targets and therapeutic opportunities. Mendelian randomization (MR) analysis can be used to assess the causal effects of exposures on outcomes with single nucleotide polymorphisms (SNPs) derived from GWAS as genetic tools, thus helping to identify possible therapeutic targets for different diseases. Unlike observational studies, the genetic instruments in MR are randomly assigned during meiosis, minimizing confounding factors and improving the reliability of causal inferences. MR analysis is extensively used in drug repurposing and drug target development. MR can discover possible therapeutic targets for various conditions, such as Alzheimer’s disease (AD) and multiple sclerosis, due to the progress in high-throughput genomic technologies and proteomics for plasma and cerebrospinal fluid (CSF) [19,20]. However, there are only a few MR researches using GWAS and protein quantitative trait loci (pQTL) data related to NMOSD.

Therefore, this study aimed to identify plasma and CSF proteins that may serve as possible drug targets for NMOSD. This research was also particularly important given the challenges faced in drug development, including cumbersome development processes, high costs, and increased failure rates. By focusing on potential drug targets, our work aims to optimize the drug development process and thereby improve the chances of successful treatments for NMOSD.

## Materials and methods

This study comprised three sequential phases: (1) instrument selection from proteome-wide datasets, (2) causal effect estimation using NMOSD GWAS, and (3) multi-dimensional validation. All analyses utilized publicly available summary statistics without direct sample-level data access.

### CSF and plasma pQTL

CSF pQTL data were obtained from the Yang et al. study [21], with 274 pQTLs for 184 CSF proteins. The inclusion criteria for pQTLs were: (i) genome-wide significance (*p* < 5 × 10⁻⁸); (ii) independence based on a linkage disequilibrium (LD) threshold of r² < 0.001; (iii) cis-acting pQTLs; and (iv) localization outside the major histocompatibility complex (MHC) region (chr6, 26–34 Mb). A total of 154 cis-pQTLs (corresponding to 154 proteins) were identified (S1 Table). Plasma pQTL data were obtained from the Zheng et al. study [22], which combined data from five previously published GWAS [23–26]. The same inclusion criteria above were used and 738 cis-acting SNPs related to 734 proteins were identified (S2 Table). The original documentation was validated to guarantee data veracity. These datasets were prioritized based on: (a) Sample size >10,000 for main analysis, (b) Protein coverage (>154 CSF/734 plasma proteins), and (c) Availability of allele frequency and LD reference panels.

Furthermore, plasma pQTL data were obtained from two recent studies for external validation (Ferkingstad et al. [27], which assessed 4,907 plasma proteins in 35,559 participants; and Pietzner et al. [28], which analyzed 4,775 plasma proteins in 10,708 participants).

Finally, the equivalent human genome construct was used as a reference for any absent information in the QTL GWAS summary statistics, including impact allele frequencies. All secondary analyses used fully anonymized, publicly accessible summary statistics from original publications. All analyses utilized open-access GWAS summary statistics under CC-BY 4.0 licenses, with original data reuse authorization confirmed through NHLBI/FNIH frameworks.

### Summary statistics of GWAS for NMOSD

The GWAS data for various antibody subtypes of NMOSD were obtained from a whole-genome sequencing study, which included 132 patients diagnosed with AQP4-IgG-positive NMOSD and 1,244 controls [29]. We rigorously applied the 2006 Wingerchuk diagnostic criteria [30], requiring optic neuritis, transverse myelitis, and ≥2 supportive features (longitudinally extensive lesions, non-MS brain MRI, or AQP4-IgG seropositivity).

### Statistical analysis

#### 1 ) MR analysis.

MR analysis was performed using the “TwoSampleMR” tool (https://github.com/MRCIEU/TwoSampleMR), plasma and CSF proteins as exposures and NMOSD as the result. The Wald ratio approach was utilized for proteins with a single available pQTL. Heterogeneity analysis and inverse-variance weighted MR (MR-IVW) were used when two or more genetic tools were available [31]. The odds ratios (ORs) for elevated NMOSD risk were determined per one standard deviation (SD) rise in plasma protein levels and per tenfold increase in CSF protein levels.

Multiple testing in the primary analysis was regulated via false discovery rate (FDR) correction, using a crucial *p*-value of 0.05 to prioritize results for further investigation. MR external validation was conducted only for proteins initially identified, with a significance level of *p* < 0.05. Preliminary findings were validated using a consistent variant methodology with the same SNPs as genetic instruments (as in the primary analysis) and a stringent variant methodology with genome-wide important SNPs as genetic instruments.

#### 2 ) Reverse causality detection.

A total of 1370 genetic instruments for NMOSD were selected from the GWAS of Estrada’s work [29] for bidirectional MR analysis to identify possible reverse causation using the same screening criteria for pQTLs [32]. Three earlier investigations provided the full summary data for proteins [21,24,27]. Effect estimates were evaluated using the weighted median, simple mode, MR-Egger, MR-IVW, and weighted mode approaches. The directionality of the protein-NMOSD interaction was verified via Steiger filtering [33]. *p* < 0.05 was considered statistically significant.

#### 3 ) Bayesian co-localization analysis.

Bayesian co-localization analysis was conducted using the “coloc” software (https://github.com/chr1swallace/coloc) (with default options) to evaluate the likelihood that two characteristics share the same causal variation. Bayesian co-localization analysis estimates the posterior probabilities of five hypotheses regarding whether two traits share a causal variant as previously described [26]. Herein, the posterior probability for hypothesis 3 (PPH_3_), which states that proteins and NMOSD are related to the area via distinct variations, and hypothesis 4 (PPH_4_), which states that proteins and NMOSD are associated with the area through a shared variant, was assessed. Genes with PPH_4_ > 80% (as assessed by at least one method) were classified as having co-localization evidence via the coloc.abf and coloc.susie algorithms [31,34].

#### 4 ) Phenotype scanning.

Phenotype scanning was conducted using the “Phenoscanner” to determine if the detected pQTLs were significantly correlated with other traits and whether they displayed pleiotropy [35]. Data were obtained from the plasma proteome GWAS of Ferkingstad’s work [27]. SNPs were classified as pleiotropic if they matched the following criteria: genome-wide relevance in European populations (*p* < 5 × 10⁻⁸) and association with any recognized risk factors for NMOSD, such as proteins, metabolic traits, or clinical phenotypes. The LD r^2^ between the pQTLs of the proteins that were prioritized was determined to find possible linkage associations.

#### 5 ) Comparison analysis and protein-protein interactions (PPI) network.

A weak association may exist between pQTLs discovered in plasma and CSF due to the blood-brain barrier (BBB). Spearman correlation analysis was performed to assess the relationship between the effect estimates from MR analyses for shared pQTLs in plasma and CSF based on diverse *P*-value criteria, evaluating whether the association changed with increasing relevance levels.

The PPI network of proteins related to the risk of NMOSD (*p* <0.05) was investigated to examine interactions among prioritized proteins and determine if proteins in plasma data may interact with those in CSF data. Additionally, three disease-modifying therapies for NMOSD and their corresponding targets were collected based on a recent review and data from the DrugBank database (https://www.drugbank.ca) to investigate interactions between these NMOSD-associated genes and existing potential drug targets [36]. The current pharmaceuticals that target the identified pathogenic proteins were also investigated. PPI analyses were performed using version 11.5 of the Search Tool for the Retrieval of Interacting Genes (STRING) database (https://string-db.org/), with a minimum interaction score threshold of 0.4 [37]. Moreover, the Wald ratio technique and coloc.abf algorithm was used for MR analyses and Bayesian co-localization, respectively, using prioritized proteins as both exposure and outcome factors. Potential interactions and potential co-localization were defined by an MR *P*-value < 0.05 and PPH_4_ > 0.8, respectively.

**Fig 1:**
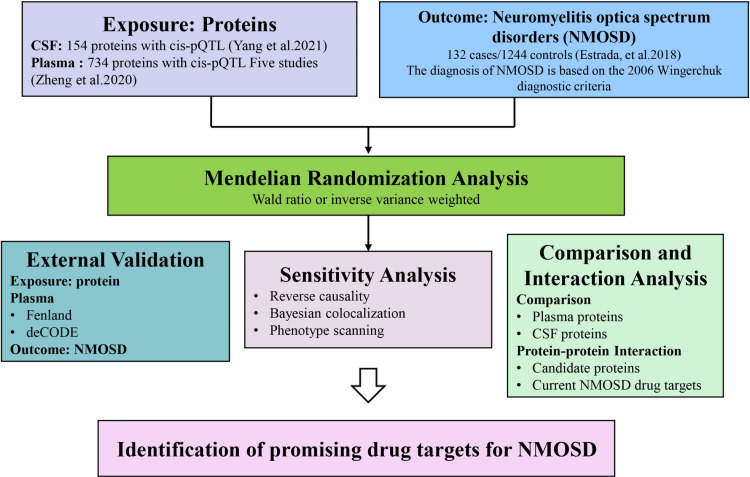
Overview of the study design aimed at identifying plasma and brain proteins that are causally linked to NMOSD.

Briefly, pQTL data for plasma and CSF proteins were combined with GWAS data for NMOSD to identify possible pathogenic proteins. The proteins were confirmed using external GWAS data. Bayesian co-localization analysis, reverse causality testing, and phenome-wide scans were used for additional validation. Enrichment analyses of phenotypes, diseases, and genes were conducted to elucidate potential mechanisms of action. These pathways were further examined by constructing an interaction network with the identified potential drug targets. The research design is shown in Fig 1.

## Results

### Proteome screening for causal proteins associated with NMOSD

**Fig 2:**
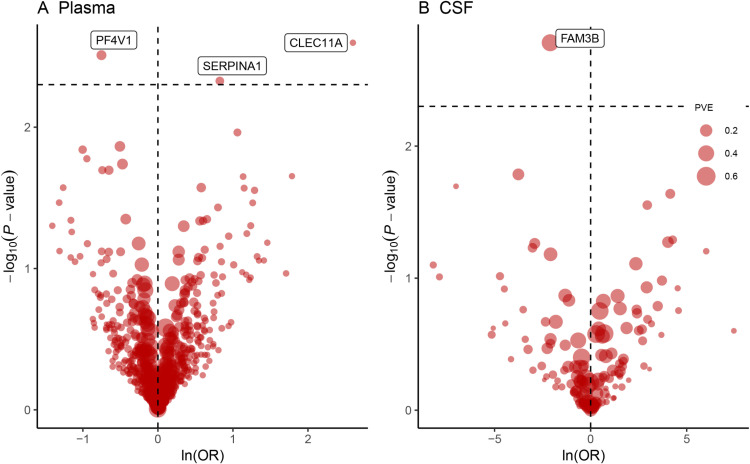
A volcano plot was created to pinpoint 4 proteins as potential targets for NMOSD from a total of (A) 734 plasma proteins and (B) 151 cerebrospinal fluid (CSF) proteins, utilizing the Wald ratio or inverse variance weighting approach. CSF: cerebrospinal fluid, PVE: proportion of variance explained.

**Fig 3:**
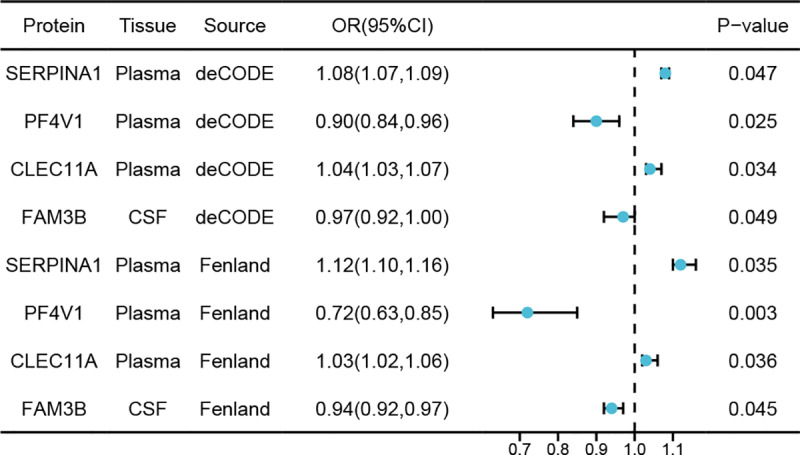
An external validation process was performed in a forest plot to examine the causal link between 4 candidate proteins and NMOSD.

MR analysis discovered four protein-NMOSD associations based on an FDR significance level (*p* < 0.05) (Table 1 and Fig 2A and B). The protein-NMOSD associations included Serine Peptidase Inhibitor, Clade A, Member 1 (SERPINA1), Platelet Factor 4 Variant 1 (PF4V1), and C-type lectin domain family 11 member A (CLEC11A) in plasma, as well as Family with Sequence Similarity 3 Member B (FAM3B) in CSF. Notably, increased PF4V1 levels (OR = 0.47; 95% CI, 0.29–0.78; *p* = 3.39 × 10^−3^) and FAM3B levels (OR = 0.12; 95% CI, 0.03–0.45; *p* = 1.65 × 10^−3^) were associated with a decreased risk of NMOSD. In contrast, elevated SERPINA1 levels (OR = 2.28; 95% CI, 1.29–4.04; *P*= 4.71 × 10^−3^) and CLEC11A levels (OR = 1.345; 95% CI, 1.29–4.04; *p* = 4.71 × 10^−3^) were related to an increased risk of NMOSD. The proteins showed no heterogeneity in the primary analysis. Furthermore, external validation of potential NMOSD drug targets was performed using deCODE and Fenland, which confirmed that the potential future drug targets were related to NMOSD (Fig 3).

**Table 1:** MR results for plasma and CSF proteins markedly linked to NMOSD after FDR-adjusted.

Tissue	Protein	UniProt ID	SNP ^a^	Effect allele	OR (95% CI) ^b^	*p* value	PVE	F statistics	Author
Plasma	SERPINA1	E9KL23; P01009	rs2749534	G	2.28 (1.29, 4.04)	4.71e-03	5.94%	62.99	Suhre
Plasma	PF4V1	P10720	rs941758	A	0.47 (0.29, 0.78)	3.09e-03	10.34%	380.79	Sun
Plasma	CLEC11A	Q9Y240	rs13866	C	13.45 (2.49, 72.61)	2.53e-03	1.08%	35.08	Emilsson
CSF	FAM3B	P58499	rs2838014	G	0.12 (0.03, 0.45)	1.65e-03	46.48%	725.15	Yang

a All SNPs used were cis-acting.

b As the levels of plasma protein increased by one standard deviation, or the levels of CSF protein increased by 10-fold, the risk of NMOSD also increased.

### Sensitivity analysis

Four proteins, including PF4V1, FAM3B, SERPINA1, and CLEC11A, were identified as potential drug targets for NMOSD via MR analysis. Various sensitivity analyses, including bidirectional MR, Bayesian co-localization analysis, and phenotypic evaluations, were conducted to confirm the relationship between NMOSD risk and the proteins. The initial MR Steiger filter showed that PF4V1 (*p* = 8.24×10^-19^), FAM3B (*p* = 2.76×10^-13^), SERPINA1 (*p* = 2.37×10^-27^), and CLEC11A (*p* = 3.61×10^-12^) were significantly correlated with NMOSD risk. Follow-up MR analyses combined with Steiger filtering found no signs of reverse causation for the proteins in the initial MR analysis (Table 2). Additionally, the co-localization study indicated that PF4V1 (PPH_4_ = 0.934), FAM3B (PPH_4_ = 0.916), SERPINA1 (PPH_4_ = 0.978), and CLEC11A (PPH_4_ = 0.895) shared variants with NMOSD (Table 2). The associations of these drug targets

**Table 2:** Sensitivity analysis on 4 potential causal proteins.

Tissue	Protein	UniProt ID	Steiger filtering	Colocalization PPH4 (coloc.abf)	Previously reported using MR
Plasma	SERPINA1	E9KL23; P01009	True (2.37×10^–27^)	0.978	increasing anxiety risk (PMID: 38525495)
Plasma	PF4V1	P10720	True (8.24×10^–19^)	0.934	N/A
Plasma	CLEC11A	Q9Y240	True (3.61×10^–12^)	0.895	increasing xanthelasma palpebrarum risk (PMID: 38601164)
CSF	FAM3B	P58499	True (2.76×10^–13^)	0.916	N/A

with other diseases reported through MR methods are summarized in Table 2. Chan et al. have reported that SERPINA1 can increase the risk of anxiety [38]. Lin et al. also found that higher CLEC11A levels are related to an increased risk of xanthelasma palpebrarum [39].

**Fig 4:**
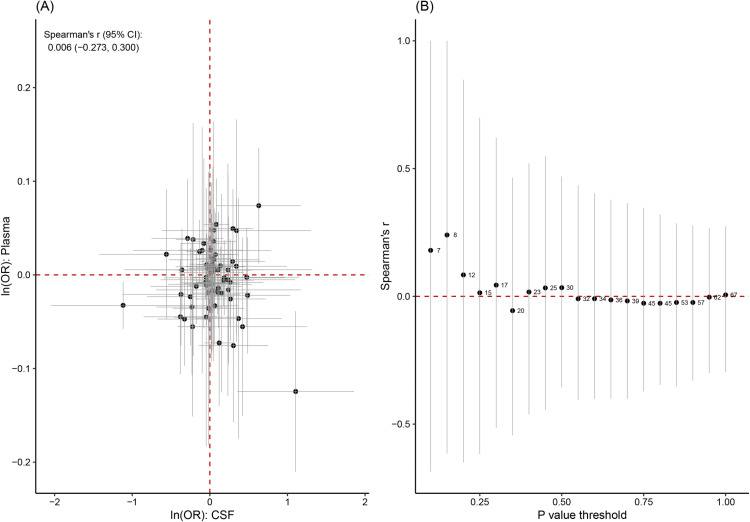
Comparative analysis of MR estimates from the plasma proteome versus the brain proteome. (A) Correlation analysis was conducted on the 67 proteins that overlapped between plasma and brain. The gray lines, both horizontal and vertical, indicate 95% confidence intervals for MR estimates. (B) The Spearman correlation coefficient was computed using various P value thresholds to incorporate MR estimates. The numbers next to the black point denote the count of overlapping proteins.

A Spearman correlation coefficient of 0.006 (95% CI: -0.273–0.300) was calculated when evaluating the causal proteins in CSF and protein-protein interactions. The analysis included 67 proteins without a defined *p*-value threshold. Nonetheless, significant correlations were detected even when various *P*-value thresholds were applied (Fig 4). The interactions among these causal proteins in plasma and CSF were then explored (S1 Fig).

### Linking Potential Drug Targets to Existing NMOSD Treatments

STRING database analysis under high-confidence thresholds (interaction score >0.7, experimental evidence only) identified a functionally enriched network of SERPINA1, CP, CTSC, and TF (FDR-corrected *p* <0.05), with text mining confirming SERPINA1-PF4V1 co-regulation. Therefore, we selected the above proteins for drug target screening.

Druggability assessment using DrugBank (v5.1.9) prioritized SERPINA1, CP, and TF as direct targets of 3 FDA-approved or experimental drugs, including alpha-1 antitrypsin replacement therapy (DB00058), ferrous gluconate (DB14488), and iron-modifying deferasirox (DB14489) (**Table 3**). Targeting drugs for CTSC, PF4V1 have not yet been discovered.

**Table 3:** Druggability of proteins potentially causally associated with NMOSD.

Target	Gene	Uniprot ID	Mediciation	Actions	Data source
Alpha-1-antitrypsin	SERPINA1	P01009	Mercaptoethanol	inhibitors	Drugbank
Ceruloplasmin	CP	P00450	Ferrous gluconate	substrate	Drugbank
Serotransferrin	TF	P02787	Ferrous succinate	substrate	Drugbank

## Discussion

Although some progress has been made in exploring treatment methods for NMOSD, current treatment strategies are unsatisfactory and face numerous challenges. In this research, the relationship between proteins in plasma and CSF with the risk of NMOSD was evaluated to detect potential therapeutic targets for NMOSD treatment. Three plasma proteins and one CSF protein associated with NMOSD, which could serve as potential drug targets, were identified. Notably, distinct proteins were identified in plasma and CSF, with no significant correlation, possibly due to the influence of the BBB. Although these are preliminary findings, the results suggest that both plasma and CSF may be effective for detecting NMOSD-related proteins. The proteins are potential therapeutic targets for NMOSD treatment.

The progress in human proteomics has improved drug development. In this study, possible treatment targets for NMOSD were identified using an integrated strategy combining MR with co-localization analysis. Moreover, the methodology was used to assess proteins with causal effects on NMOSD and convert previous GWAS discoveries into therapeutic applications. The causal relationships identified through MR may be affected by various variables, including horizontal pleiotropy, reverse causation, and genetic confounding caused by LD. As a result, the directionality of causal inference was evaluated using Steiger filtering. Furthermore, cis-pQTLs were selected as IVs to reduce biases caused by horizontal pleiotropy, due to their direct involvement in the transcription or translation of the associated genes. LD-related biases were reduced via Bayesian co-localization analysis using a PPH_4_ threshold of 0.8 to identify proteins that share variants with NMOSD, thereby enhancing the robustness of the analysis. To the best of our knowledge, this is the first work combining proteomics data from plasma and CSF, as well as combining two-sample MR with Bayesian co-localization to identify random proteins related to NMOSD. Herein, four potential therapeutic targets for NMOSD were identified (SERPINA1, PF4V1, and CLEC11A in plasma; FAM3B in CSF). Further analysis utilizing similar techniques in the deCODE and Fenland cohorts confirmed the association between these proteins and NMOSD.

Specifically, elevated plasma SERPINA1 levels were associated with an increased risk of NMOSD. The SERPINA1 gene encodes a serine protease inhibitor, Alpha-1 antitrypsin (A1AT), belonging to the serpin superfamily [40]. SERPINA1 primarily exerts anti-inflammatory effects by inhibiting proteases, such as neutrophil elastase and trypsin. However, some studies have suggested that SERPINA1 deficiency may increase local inflammation under certain pathological conditions [41]. Moreover, the role of SERPINA1 in immune response regulation has attracted much attention in recent years. SERPINA1 modulates immune cell functions by interacting with various cytokines and cell surface receptors [42]. For instance, SERPINA1 can affect tumor necrosis factor α (TNF-α) signaling, which is critical in autoimmune diseases [43]. Additionally, the methylation levels of SERPINA1 are positively correlated with IL-1β levels [40]. Notably, IL-1β exerts pro-inflammatory effects in NMOSD [44–46]. In conclusion, the interplay between the anti-inflammatory and pro-inflammatory functions of SERPINA1 plays a crucial role in various physiological and pathological processes, offering new insights into its role in NMOSD.

PF4V1, also known as CXCL4L1, is involved in angiogenesis, inflammation, and tumor [47]. Also, PF4V1 exhibits potent anti-angiogenic properties, effectively inhibiting tumor growth. PF4V1 also preserves the integrity of the blood-retinal barrier in diabetes patients. PF4V1 can regulate renal inflammation by inhibiting the activation of inflammatory genes through mechanisms involving IκBα and SMAD7 [48]. Recent findings have revealed that PF4V1 can induce monocyte differentiation into a macrophage phenotype distinct from the conventional M1 and M2 types [49]. Levels of inflammatory factors are increased in patients with Nasu-Hakola disease, while PF4V1 expression is significantly decreased, underscoring its anti-inflammatory role [50]. Herein, higher plasma levels of PF4V1 were associated with a reduced risk of NMOSD, possibly through anti-inflammatory effects. In addition, studies have demonstrated that the interaction between neurons and glial cells plays an important role in NMOSD development [51]. These findings indicate that PF4V1 may exert its therapeutic effects by modulating the intercellular signaling between these cells. Furthermore, studies on other disease targets, such as the role of the translocator protein (TSPO) in glioblastoma [52] and the potential role of GPR35 in diabetes and hypertension [53], provide valuable references for understanding the mechanism of action of PF4V1 in NMOSD.

Furthermore, elevated plasma levels of CLEC11A were associated with an increased risk of NMOSD. CLEC11A is a glycoprotein essential for osteoblast maturation and hematopoiesis [54]. Additionally, CLEC11A is associated with the pathogenesis of several tumors, such as lung cancer, gastrointestinal tumors, multiple myeloma, and leukemia [55]. Recent research has also indicated that CLEC11A participates in the development of several neurological diseases. Notably, levels of CLEC11A are elevated in the CSF of patients with chronic inflammatory demyelinating polyneuropathy and in the blood of patients with chronic spinal cord injuries over time, suggesting its potential involvement in chronic neurodegenerative processes [56,57]. Furthermore, CLEC11A expression in the CSF is increased in patients with trigeminal neuralgia, indicating that CLEC11A may promote peripheral nerve inflammation, demyelination, and neurodegeneration [58]. These findings indicate that CLEC11A is a promising therapeutic target for NMOSD.

Moreover, elevated CSF levels of FAM3B were associated with a reduced risk of NMOSD. FAM3B, a cytokine-like protein also known as pancreatic-derived factor, was found in pancreatic α and β cells in 2002 [59]. FAM3B is also expressed in most normal human tissues and various cancers [60]. The varied expression patterns and biological roles of FAM3B in distinct cancer types may be due to the alternative splicing [61]. As a result, FAM3B is associated with several significant disorders, including diabetes, AD, and cancer [62]. However, only a few studies have detected FAM3B levels in CSF. FAM3B is also expressed in central neurons, where it can inhibit TNF-α-induced cell death [63]. TNF-α is a key inflammatory factor involved in the progression of NMOSD, indicating that FAM3B may exert anti-inflammatory effects and reduce neuronal apoptosis, potentially mitigating NMOSD progression. Nonetheless, future studies should investigate the impact of FAM3B expression levels in CSF on NMOSD pathogenesis.

Both this study and the recent MR study [64] aim to identify therapeutic targets for NMOSD, yet provide complementary evidence at distinct biological layers. While our proteomic analysis reveals coordinated regulation networks of peripheral pro-/anti-inflammatory proteins interacting with the central nervous system, the published study proposes novel targets such as NEU1 through gene expression regulation. These findings collectively advance multi-dimensional perspectives on target discovery, though their therapeutic value requires validation via multi-omics integration and clinical trials. Notably, our study’s target specificity may be limited by unexamined associations with other autoimmune diseases, whereas the reliance on eQTL data in Zhang et al. introduces potential tissue-specific biases between gene expression and protein function. Together, we promote multiple perspectives on NMOSD target discovery, but further integration of multiple omics data and clinical validation is needed to clarify therapeutic value.

However, this study has some limitations. First, only cis-pQTLs were selected as IVs, and most proteins had fewer than three SNPs, limiting the application of certain MR methods, such as MR Egger intercept test, Cochran’s Q test, and MR-PRESSO for multivariable residuals and outliers. Second, only individuals of European heritage were included to reduce bias resulting from demographic stratification, indicating that the results may not apply to other ethnicities. Therefore, further studies should include more GWAS data on NMOSD from different populations. Therefore, this study provides essential insights into the genetic underpinnings of NMOSD.

## Conclusions

In conclusion, plasma proteins SERPINA1, PF4V1, CLEC11A, and CSF protein FAM3B, determined by genetic factors, are causally related to the risk of NMOSD. These proteins are associated with NMOSD and could serve as potential future drug targets. Nonetheless, further investigation should explore the potential mechanisms related to these proteins in NMOSD.

## Supporting information

S1 Fig The interactions among these causal proteins in plasma and CSF. (TIF)

S1 Table A total of 154 cis-pQTLs (corresponding to 154 proteins) were identified. (XLSX)

S2 Table 738 cis-acting SNPs related to 734 proteins were identified. (XLSX)
