# Supplementary figures and images for: Potential drug targets for Neuromyelitis optica spectrum disorders (NMOSD): A Mendelian randomization analysis
Source: PLoS One. 2025 Apr 28;20(4):e0322098. doi: 10.1371/journal.pone.0322098 (PMC12083898; doi:10.1371/journal.pone.0322098)

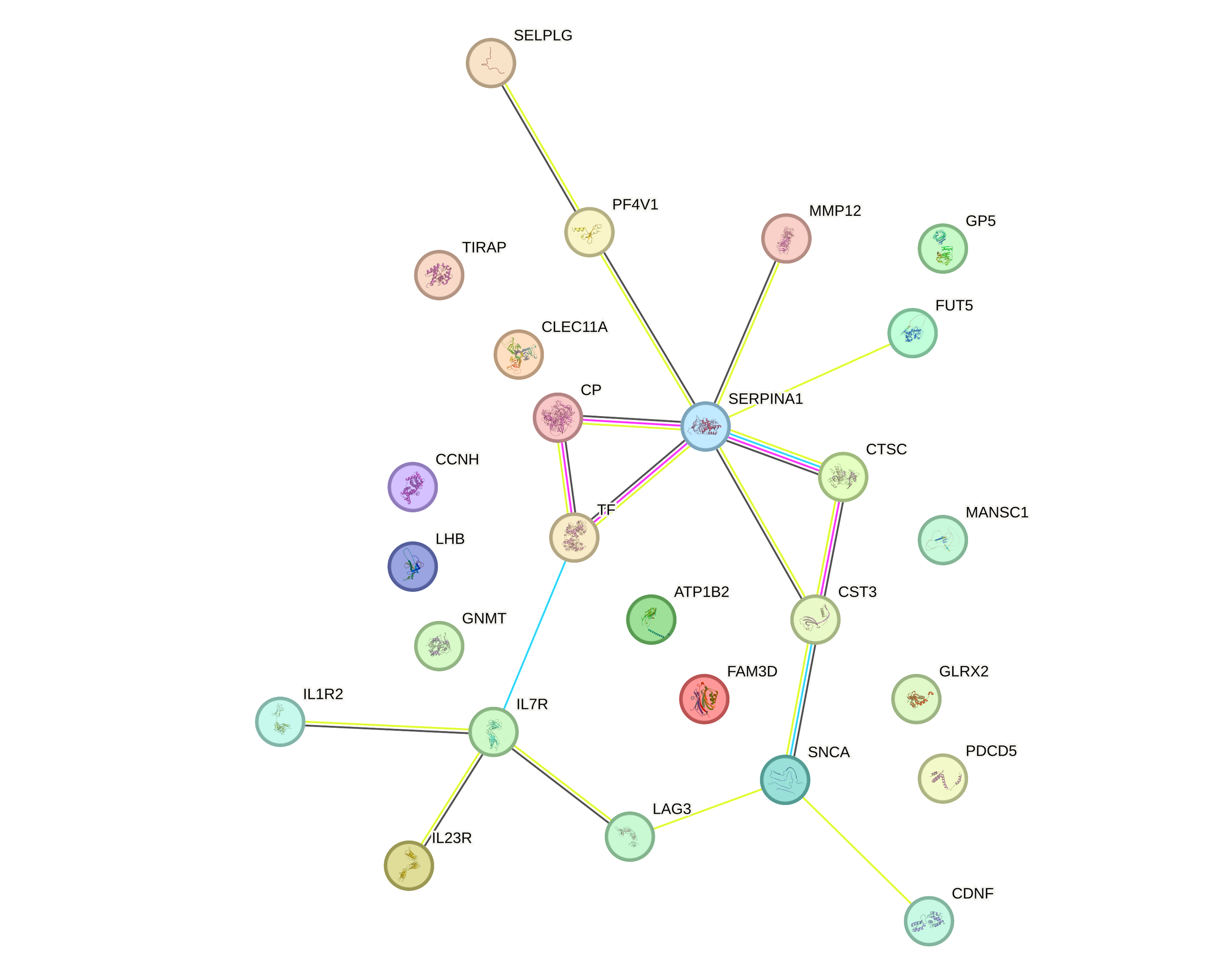

Supplement: S1 Fig — (TIF) [file pone.0322098.s001.tif]
